# One case of swine hepatitis E virus and porcine reproductive and respiratory syndrome virus Co-infection in weaned pigs

**DOI:** 10.1186/1743-422X-10-341

**Published:** 2013-11-19

**Authors:** Jingjing Mao, Yue Zhao, Ruiping She, Peng Xiao, Jijing Tian, Jian Chen

**Affiliations:** 1 Department of Veterinary Pathology, Key Laboratory of Zoonosis of Ministry of Agriculture, China Agricultural University, Beijing 100193, China

**Keywords:** Weaned pig, HEV, PRRSV, Co-infection

## Abstract

**Background:**

Using various methods, we analyzed the cause of death among weaned pigs from a pig farm in Hebei Province, China. All 300 piglets (100% fatality) were identified as moribund, with death occurring within 1 month from the onset of clinical signs.

**Results:**

A single case exhibited obvious hemorrhagic necrotic changes with massive lymphocytic infiltration in multiple organs, in particular the liver, lungs and intestines. Dysplasia and lymphocyte deterioration were common in lymphatic organs. No visible bacterial colonies from liver and spleen were observed in nutrient, MacConkey, and blood agar plates. Using polymerase chain reaction techniques for this case, we attempted to detect a number of epidemic swine viruses in spleen and liver, including PRRSV, CSF, HEV, and PCV2. We found that this sample was positive for the presence of HEV and PRRSV.

**Conclusions:**

We have detected HEV and PRRSV co-infection in one piglet. Severe pathologic changes were observed. The high mortality of weaned pigs which showed the similar clinical syptom was possibly a result of HEV and PRRSV co-infection, which has rarely been reported previously. We speculated that co-infection with PRRSV and HEV might lead to more serious problems.

## Background

Over recent years, pig-related meat production in China has been subject to contamination with epidemic diseases. Infection with various pathogens has resulted in high morbidities and mortalities among pig farms. PRRS is the primary disease resulting in high mortalities and massive economic losses. HE is another epidemic disease that has been identified in pig farms. It is a zoonotic and foodborne-transmitted disease with a range of animal reservoirs other than humans.

Swine herds exhibiting reproductive failure and respiratory disease caused by PRRS were first identified in the mid-west of the USA from 1987–1988. The disease then rapidly spread throughout swine-growing regions of the USA and Europe, followed by the rest of the world [1]. In China, PRRS was first identified in an intensive pig farm in northern China at the end of 1995 [2]. In the subsequent 10 years, PRRS became prevalent among Chinese pigs [3], with an average seropositive rate of 11–39%. In China, PRRS is characterized by respiratory disorders and reproductive failure, such as dead or mummified fetuses, irregular abortions in sows and stillborn or weak-born pigs. The occurrence of PRRS mixed infection with other diseases such as CSF and PCV2 is now common and serious in China [4]. The etiological agent of PRRS is porcine reproductive and respiratory syndrome virus (PRRSV).

HE is usually an acute, self-limiting disease caused by hepatitis E virus (HEV). HEV is non-enveloped, with a positive-sense, single-stranded RNA genome of approximately 7.2 kb, and is transmitted *via* the fecal-oral route [5,6]. There are at least four genotypes of HEV, with genotypes 3 and 4 known to infect animals, and thought to be zoonotically transmitted. Balayan et al. were the first to report the experimental infection of domestic swine with an Asian strain of human HEV in 1990 [7]. Meng et al. then discovered a novel virus in naturally infected pigs that resembled a strain of human HEV; this was designated swine HEV [8]. HE is prevalent throughout China, with both genotypes 3 and 4 found in humans and swine. Genotype 4 is the primary swine genotype found throughout most of China [9].

Infection of pigs with multiple diseases is common, however co-infections involving PRRSV and HEV have rarely been reported. The objective of our study was to analyze the cause of death in a large number of weaned piglets from China, and provide a new perspective for clinical research.

## Case presentation

From February to March 2011, an outbreak of an unknown disease occurred at a medium scale pig farm with 300 piglets in Hebei province, China. The affected animals were weaned piglets that were around 40 days old. Animals presented with symptoms of anorexia, asthma, high fever (about 41°C) and significant neurological symptoms. The disease was characterized by acute onset and short duration. During the initial stages of the disease, pigs failed to respond to antibiotic treatment. During one month, all 300 sick piglets were dead and the fatality rate was 100%. Immunization with a PRRSV vaccine had been conducted at this farm prior to the disease outbreak.

A single dead piglet was examined and necropsied. No obvious hemorrhaging was observed on the surface of the piglet corpse. The right ventricle was dilated, and the myocardium was pale and flabby. Congestion, hemorrhage and necrosis could be seen in large local areas of the lung, and a small volume of a white foam-like murky liquid was present inside the trachea. The kidney was pale and visible hemorrhaging was observed between the cortex and medulla on the sagittal surface. The liver was enlarged, and color on the surface was light to dark. Spleen and mesenteric lymph nodes were slightly swollen. Small local hemorrhagic ulcers were seen on the gastric mucosa, along with mesenteric vascular engorgement. The intestinal mucosa was hyperemic and appeared to be flushed.

**Figure 1:**
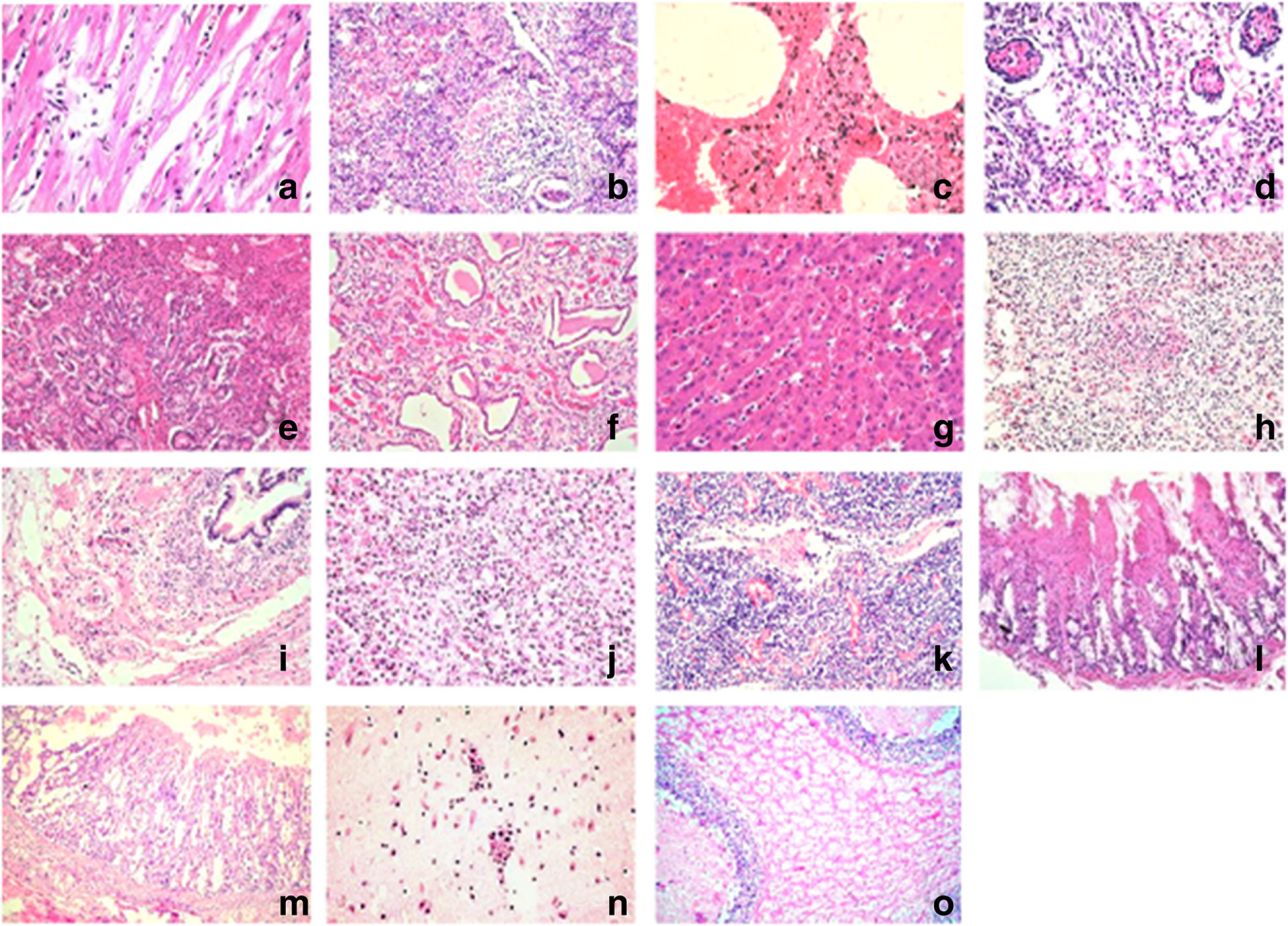
**Histological lesions in multiple organs.** Pathological changes were characterized by hyperemia, hemorrhaging, necrosis and inflammatory infiltration. Staining was conducted with hematoxylin and eosin. **(a)** Disarrayed or dissolved myocardia. **(b, c)** Lung with extensive hemorrhaging, lymphocyte infiltration and exfoliated alveolar epithelium cells inside the bronchioles and alveoli. **(d, e, f)** Necrosis and hemorrhaging in the kidney. **(g, h, i)** Liver exhibiting hepatic necrosis and lymphocyte infiltration. **(j)** Dysplasia of lymphoid follicles in the spleen. **(k)** Lymph nodes with congested capillaries. **(l, m)** Coagulation necrosis and abruption of intestinal villi. **(n, o)** Perivascular cuffs in cerebrum and liquefaction necrosis in cerebellum. Magnification was 400× **(a, c, g, j, n)**, 200× **(b, d, e, f, h, i, k, o)**, or 100× **(l, m)**.

Pathological changes in various tissues were determined by microscopy. In the heart, the myocardial interstitium broadened with mild edema. The myocardium was generally granular, degenerated, disarrayed or dissolved (Figure 1a). A small number of lymphocytes had infiltrated among the myocytes. For the lung, different pulmonary lobules presented different degrees of pathological changes. In some pulmonary lobules, alveoli were abundantly filled with cells, erythrocytes and exudates, and the normal histological structure of the lung was severely compromised. Exfoliated alveolar epithelium cells were accumulated inside the bronchioles (Figure 1b); in some pulmonary lobules, extensive hemorrhaging was observed in the alveoli and interstitium (Figure 1c). In other areas, the alveolar walls were thickened, with substantial lymphocyte infiltration. Infiltration by numerous inflammatory cells and pink liquid exudates in the interlobular arteries were commonly seen. In the kidney, some of the cortical glomeruli were necrotic and contained erythrocytes. Large numbers of tubular epithelial cells were vacuolated, exfoliated and necrotic, especially at the edge of cortex (Figure 1d). A protein cast could be seen in some renal tubules (Figure 1e). In the renal junction area between the cortex and medulla, congestion and hemorrhaging was obvious (Figure 1f). Examination of the liver revealed congestion, hemorrhaging, lymphocyte infiltration and an accumulation of randomly distributed lymphocytes. There was hepatic necrosis and vacuolization in large local areas (Figure 1g, 1h). Fibrosis was apparent around the portal area, with proliferation of small bile ducts, and lymphocyte infiltration also observed (Figure 1i). Within the spleen the number of lymphocytes was decreased in the white pulp area around the central artery. The lymphoid follicles were smaller than average and subject to dysplasia (Figure 1j). The capillaries of lymph nodes were dilated and congested, and expansion of lymphatic sinuses was obvious (Figure 1k). Examination of the intestine revealed coagulation, necrosis, desquamation, and abruption of intestinal villi in some areas. Necrosis of intestinal epithelial cells was observed, as well as exposure of the lamina propria, with dense lymphocyte infiltration beyond the layer of the muscularis mucosa. There was an increase in the levels of secretion from intestinal glands (Figure 1l, 1m). We noticed that hyperplasia of gliacytes, demyelination and perivascular cuffs were visible in the cerebrum. At the molecular layer of the cerebellum, liquefaction necrosis was evident (Figure 1n, 1o).

**Figure 2:**
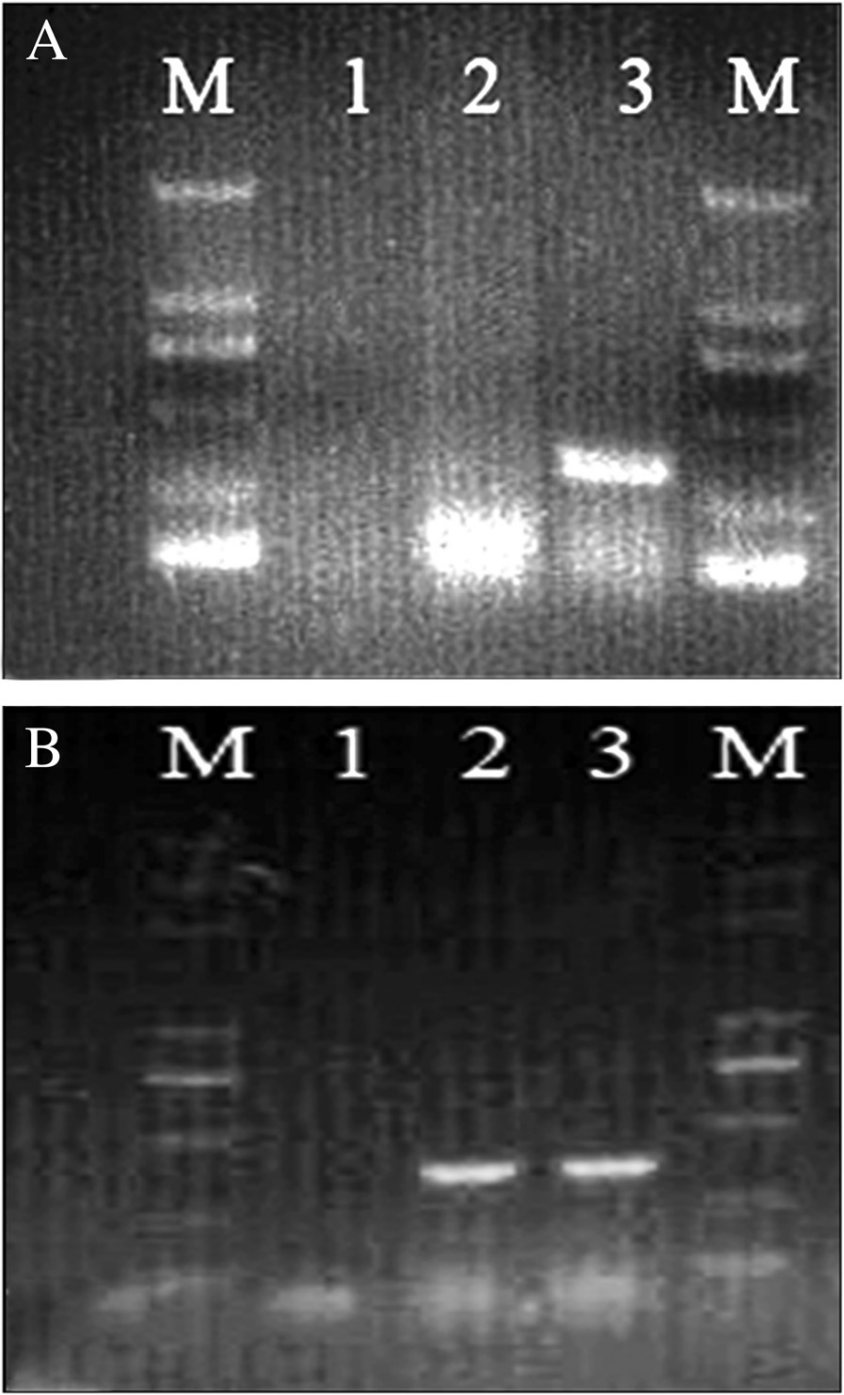
**PCR assays on liver tissues using primers specific for PRRSV and HEV. (A)** Lane M, DL2000 marker; 1, negative control; 2, lung; 3, liver. The HEV amplicon was 348 bp (arrow). **(B)** M, DL5000 marker; **1**, negative control; **2**, lung; **3**, liver. The PRRSV amplicon was 332 bp (arrow).

**Figure 3:**
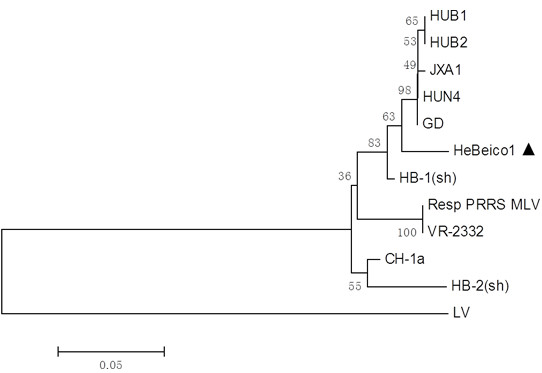
**Phylogenetic analysis based on ORF7 (332 nt) depicting the genetic relationship between our isolate from this study and isolates from across China or other countries.** A neighbor-joining tree was constructed with bootstrap values calculated from 1,000 replicates. Isolates used for comparative analysis were HUB1 (GenBAnk Accession No. EF075945), HUB2 (EF112446), JXA1 (FJ548854), HUN4 (EF635006), GD (EU825724), RespPRRS MLV (AF066183), VR-2332 (U87392), CH-1a (AY032626), and LV (M96262).

**Figure 4:**
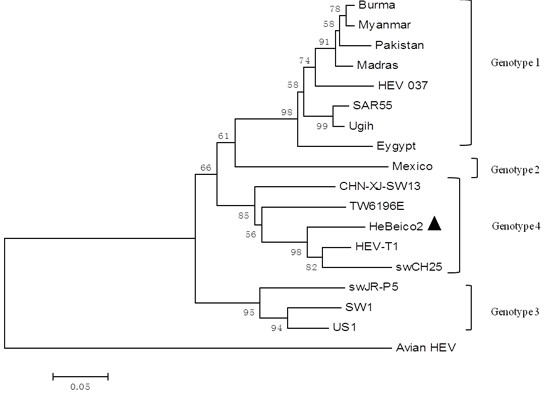
**Phylogenetic analysis based on ORF2 (348 nt) depicting the genetic relationship between our isolate from this study and isolates from across China or from other countries.** A neighbor-joining tree was constructed with bootstrap values calculated from 1,000 replicates. Isolates used for comparative analysis were Burma (GenBank Accession No. M73218), Myanmar (D10330), Pakistan (AF185822), Madras (X99441), HEV 037 (X98292), SAR55(M80581), Ugih (D11092), Eygypt (AF051352), Mexico (M74506), CHN-XJ-SW13 (GU119961), TW6196E(HQ634346), HEV-T1 (AJ272108), swCH25 (AY594199), swJR-P5 (AB481229), US2 (AF060669), US1 (AF060668), and Avian HEV (AY535004).

Using nutrient, MacConkey, and blood agar plates, there were no visible bacterial colonies. Antibiotic therapy did not affect morbidity or mortality rates. We used polymerase chain reaction (PCR) techniques to determine the presence of viruses (Table 1). We detected the presence of PRRSV, HEV, CSFV, and PCV2. PRRSV and HEV were detected in liver tissues, while liver and lung tissues were negative for CSFV and PCV2 (Figure 2) [10]. The Hebeico1 PRRSV isolate was a North American genotype, and was closely related to the HUB1, HUB2, JXA1, HUN4, GD (Figure 3). The Hebeico2 HEV isolate belonged to genotype 4 (Figure 4).

**Table 1:** Primer sequences used in PCR assays

**Primer**	**Sequence 5′-3′**	**Reference**
**PRRSV ORF7 gene**		
PRRSV-F	CCAAATAACAACGGCAAGCA	
PRRSV-R	ATGCTGAGGGTGATGCTGTGA	
**HEV ORF2 gene**		
HEV- externer primer	AATTATGCYCAGTAYCGRGTTG	[8]
HEV- externer primer	CCCTTRTCYTGCTGMGCATTCTC	
HEV- interner primer	GTWATGCTYTGCATWCATGGCT	
HEV- interner primer	AGCCGACGAAATCAATTCTGTC	
**CSFV E2 gene**		
E2- externer primer	GCATCAACCAYKGCATTCC	[11]
E2- externer primer	GTCTGTGTGGGTRATTAAGTTCCCTA	
E2- interner primer	CTRGTRACTGGGGCACAAGG	
E2- interner primer	ACCAGCRGCGAGTTGYTCTG	
**PCV2 ORF2 gene**		
PCV2.S4	CACGGATATTGTAGTCCTGGT	[12]
PCV2.A4	CCGCACCTTCGGATATACTGTC	

## Discussion

Tian et al. first reported an outbreak of highly pathogenic PRRS in China in 2006. This highly pathogenic PRRS differed from typical PRRSV, which is prevalent in the majority of China, causing high mortalities (>400,000 fatal cases). Clinical symptoms were characterized by high fever, petechiae, and erythematous blanching rash [13]. In this study, the clinical and pathological changes were consistent and similar with typical PRRS. However, some pathological damage resembled that seen for PRRS. Hemorrhaging, necrosis and lymphocyte infiltration was seen in different organs, but especially in the lungs and immune organs. Typical pathological changes such as interstitial hemorrhagic pneumonitis and depletion of splenic lymphocytes were obvious [7,13]. PRRSV comprises eight open reading frames (ORFs), with ORFs 5, 3 and 7 the most variable [14,15]. PCR results for the detection of ORF7 revealed that PRRSV was present in the liver. The Hebeico1 isolate (Figure 3) was a North American genotype that was closely related to the highly pathogenic isolates HUB1, HUB2, JXA1, HUN4, and GD. Genetic analysis and associated pathological changes indicated that our isolate likely had high pathogenicity.

For our isolate, unique symptoms were evident; there were no petechiae upon the surface of the body and the liver was severely injured. HEV is known to infect humans and pigs [8], and has four genotypes. Genotypes 1 and 2 have only been found in humans, while genotypes 3 and 4 have been recovered from both humans and pigs [16]. In the livers of pigs naturally infected or intravenously inoculated with HEV, focal lymphocyte infiltration and swollen, vacuolated hepatocytes were observed [8,17]. In our cases, we observed hepatic necrosis and vacuolization in large local areas. Fibrosis and lymphocyte infiltration were mainly observed around the portal area. We suspected HEV infection, and a PCR assay targeting HEV ORF2 showed that HEV was present in the liver. The Hebeico2 isolate belonged to genotype 4 (Figure 4), one of the most common genotypes in China [9].

We also detected other pathogens. The sick piglets failed to respond to antibiotic treatment, and no visible bacterial colonies were observed by bacterial isolation and identification. The results indicated low possibility of bacterial infection. Another two kinds of viruses PCV2 and CSFV were selected for the similar symptoms they showed and their inclination of co-infection. However, both of them failed to be detected by PCR.

**Figure 5:**
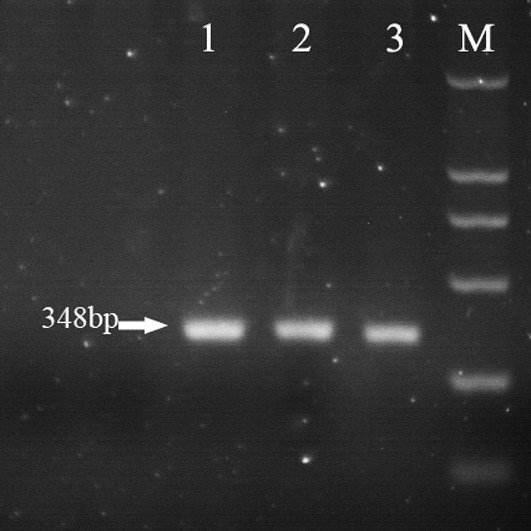
**PCR analysis of the livers from gerbils, with specific primers designed against HEV.** Lane M, DL2000 marker. **1**, **2**, and **3**, liver samples from inoculated gerbils taken at 7 days post-infection. The HEV amplicon was 348 bp (arrow).

**Figure 6:**
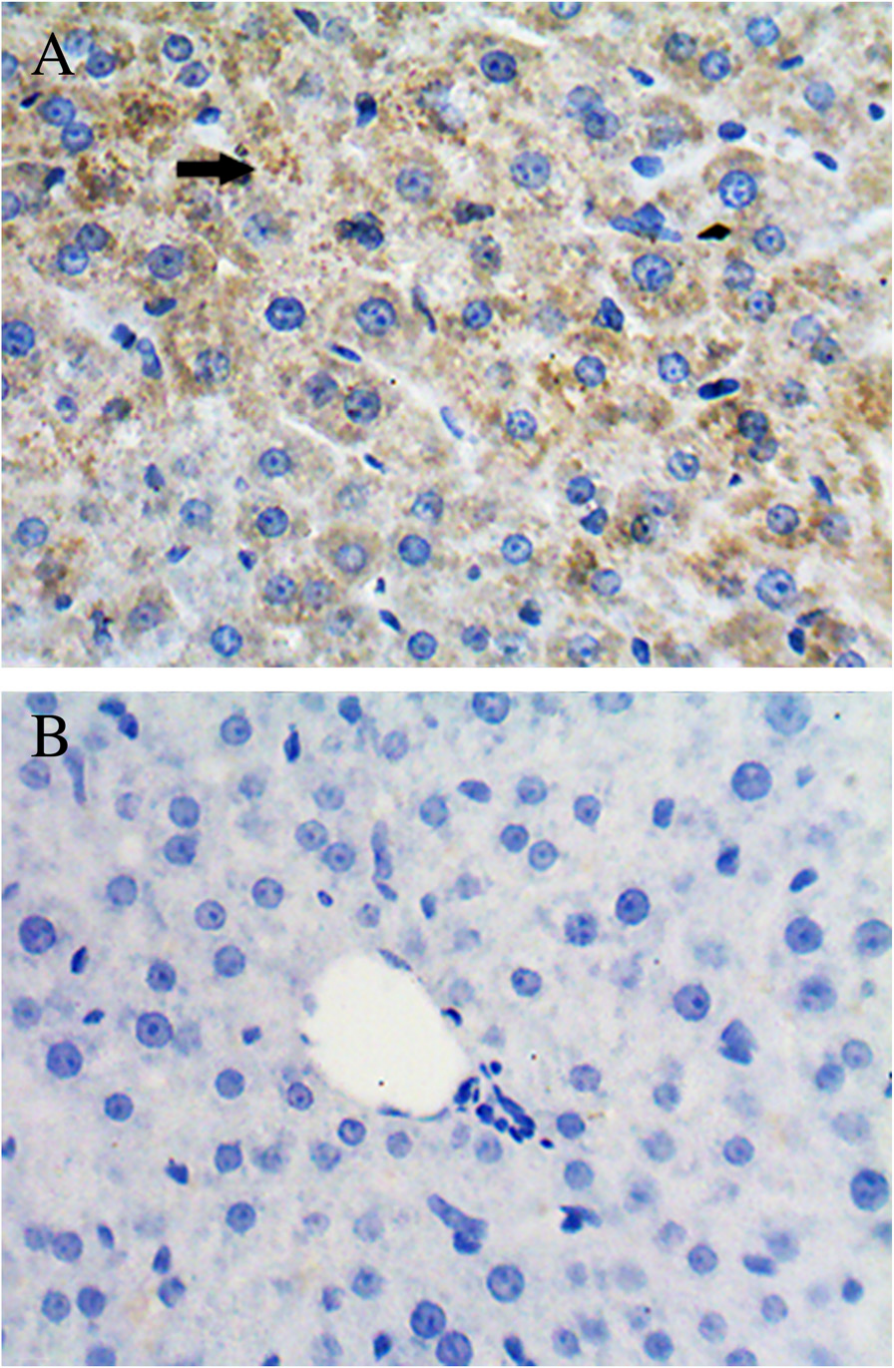
**HEV antigen detecetion in the liver of a gerbil at 7 days post-infection.** HEV antigen-positive signals (black arrow) were observed in the cytoplasm **(A)**, but not seen in the negative control **(B)**.

We used gerbils as animal models to determine if infection with the two viruses resulted in similar symptoms. We detected HEV in the livers of gerbils, using PCR, from 7 days post-infection (Figure 5), but failed to detect PRRSV. A positive signal for the HEV antigen was also be detected in the livers of gerbils using immunohistochemistry techniques (Figure 6).

Compulsory vaccination against PRRSV has been conducted in China since 2007, with the spread of PRRS being effectively, but not completely, controlled. Infectivity of HEV was very high, however accurate mortality rates remain unclear, and experimental infection with HEV did not result in death [7]. Co-infection with PRRSV and HEV was evident in our study, with all sick pigs displaying similar symptoms before dying; mortality approached 100%. We speculated that co-infection with PRRSV and HEV might lead to more serious problems.

## Conclusions

We have detected HEV and PRRSV co-infection in one weaned pig. Severe pathologic changes in this piglet and high mortality in the pigfarm indicated that HEV and PRRSV co-infection might result in serious consequences. However, whether the two viruses can interact, and the role of HEV during a PRRS infection, remains to be elucidated. Further studies are required to better understand the linked pathogenesis between HEV and PRRSV.

## Consent

Written informed consent was obtained from the farm owner for the publication of this report and any accompanying images.

## Abbreviations

HEV: Hepatitis E virus; HE: Hepatitis E; PRRSV: Porcine reproductive and respiratory syndrome virus; PRRS: Porcine reproductive and respiratory syndrome; CSF: Classical swine fever; PCV2: Porcine circovirus 2.

## Competing interests

The authors have declared no competing of interests.

## Authors’ contributions

JM, YZ and RS were responsible for the study design. JM and YZ wrote the report. JM, YZ, PX, JC did the laboratory work. JM, YZ and RS analyzed and interpreted the data. PX helped to draft the manuscript. All authors read, commented and approved the final article.

## References

[B1] Goyal SM Porcine reproductive and respiratory syndrome J Vet Diagn Invest 1993 5 656 664 10.1177/104063879300500435 8286480

[B2] Baoqing G Zhangshui C Yizhu C Isolation and Identification of Porcine Reproductory and Respiratory Syndrome (PRRS) Virus from Aborted Fetuses Suspected of PRRS 1996 Preventive Veterinary Medicine: Chinese Journal of

[B3] Zhou L Yang H Porcine reproductive and respiratory syndrome in China Virus Res 2010 154 31 37 10.1016/j.virusres.2010.07.016 20659506

[B4] Wei T-c Tian Z-j Zhou Y-j An T-q Jiang Y-f Xiao Y Hu S J-m P Hao X-f Zhang S-r TG-Z Evaluation of the pathogenicity of a highly pathogenic porcine reproductive and respiratory syndrome virus variant in piglets Agric Sci China 2011 10 1280 1291 10.1016/S1671-2927(11)60120-X

[B5] Reyes GR Purdy MA Kim JP Luk KC Young LM Fry KE Bradley DW Isolation of a cDNA from the virus responsible for enterically transmitted non-A, non-B hepatitis Science 1990 247 1335 1339 10.1126/science.2107574 2107574

[B6] Purdy MA Khudyakov YE The molecular epidemiology of hepatitis E virus infection Virus Res 2011 161 31 39 10.1016/j.virusres.2011.04.030 21600939

[B7] Balayan M Usmanov R Zamyatina N Djumalieva D Karas F Experimental hepatitis E infection in domestic pigs J Med Virol 1990 32 58 59 10.1002/jmv.1890320110 2122999

[B8] Meng XJ Purcell RH Halbur PG Lehman JR Webb DM Tsareva TS Haynes JS Thacker BJ Emerson SU A novel virus in swine is closely related to the human hepatitis E virus Proc Natl Acad Sci 1997 94 9860 10.1073/pnas.94.18.9860 9275216 PMC23282

[B9] Liu P Li L Wang L Bu Q Fu H Han J Zhu Y Lu F Zhuang H Phylogenetic analysis of 626 hepatitis E virus (HEV) isolates from humans and animals in China (1986–2011) showing genotype diversity and zoonotic transmission Infect Genet Evol 2012 12 428 434 10.1016/j.meegid.2012.01.017 22306814

[B10] Feagins A Opriessnig T Guenette D Halbur P Meng XJ Detection and characterization of infectious Hepatitis E virus from commercial pig livers sold in local grocery stores in the USA J Gen Virol 2007 88 912 10.1099/vir.0.82613-0 17325364

[B11] Chen N Hu H Zhang Z Shuai J Jiang L Fang W Genetic diversity of the envelope glycoprotein E2 of classical swine fever virus: Recent isolates branched away from historical and vaccine strains Veterinary Microbiology 2008 127 286 299 10.1016/j.vetmic.2007.09.009 17976931

[B12] Caprioli A McNeilly F McNair I Lagan-Tregaskis P Ellis J Krakowka S McKillen J Ostanello F Allan G PCR detection of porcine circovirus type 2 (PCV2) DNA in blood, tonsillar and faecal swabs from experimentally infected pigs Research in Veterinary Science 2006 81 287 292 10.1016/j.rvsc.2006.01.001 16481016

[B13] Tian K Yu X Zhao T Feng Y Cao Z Wang C Hu Y Chen X Hu D Tian X Emergence of fatal PRRSV variants: unparalleled outbreaks of atypical PRRS in China and molecular dissection of the unique hallmark PLoS One 2007 2 e526 10.1371/journal.pone.0000526 17565379 PMC1885284

[B14] Meulenberg JJM Hulst MM de Meijer EJ Moonen PLJM den Besten A de Kluyver EP Wensvoort G Moormann RJM Lelystad virus, the causative agent of porcine epidemic abortion and respiratory syndrome (PEARS), is related to LDV and EAV Virology 1993 192 62 72 10.1006/viro.1993.1008 8517032 PMC7173055

[B15] Le Gall A Legeay O Bourhy H Arnauld C Albina E Jestin A Molecular variation in the nucleoprotein gene (ORF7) of the porcine reproductive and respiratory syndrome virus (PRRSV) Virus Res 1998 54 9 21 10.1016/S0168-1702(97)00146-9 9660067

[B16] Ahmad I Holla RP Jameel S Molecular virology of hepatitis E virus Virus Research 2011 161 47 58 10.1016/j.virusres.2011.02.011 21345356 PMC3130092

[B17] Halbur PG Kasorndorkbua C Gilbert C Guenette D Potters MB Purcell RH Emerson SU Toth TE Meng XJ Comparative pathogenesis of infection of pigs with hepatitis E viruses recovered from a Pig and a human J Clin Microbiol 2001 39 918 923 10.1128/JCM.39.3.918-923.2001 11230404 PMC87850

